# Inter-comparison of quantitative imaging of lutetium-177 (^177^Lu) in European hospitals

**DOI:** 10.1186/s40658-018-0213-z

**Published:** 2018-08-02

**Authors:** Jill Wevrett, Andrew Fenwick, James Scuffham, Lena Johansson, Jonathan Gear, Susanne Schlögl, Marcel Segbers, Katarina Sjögreen-Gleisner, Pavel Solný, Michael Lassmann, Jill Tipping, Andrew Nisbet

**Affiliations:** 1 University of Surrey, Guildford, UK; 2 National Physical Laboratory, Teddington, UK; 3 Royal Surrey County Hospital NHS Foundation Trust, Guildford, UK; 4 The Royal Marsden NHS Foundation Trust, Sutton, UK; 5 University Hospital of Wϋrzburg, Wϋrzburg, Germany; 6 Erasmus University Medical Centre, Rotterdam, Netherlands; 7 Department of Medical Radiation Physics, Clinical Sciences Lund, Lund University, Lund, Sweden; 8 Motol University Hospital, Prague, Czech Republic; 9 The Christie NHS Foundation Trust, Manchester, UK

**Keywords:** Lutetium, Lu-177, SPECT/CT, Quantitative imaging, PRRT, Molecular radiotherapy

## Abstract

**Background:**

This inter-comparison exercise was performed to demonstrate the variability of quantitative SPECT/CT imaging for lutetium-177 (^177^Lu) in current clinical practice. Our aim was to assess the feasibility of using international inter-comparison exercises as a means to ensure consistency between clinical sites whilst enabling the sites to use their own choice of quantitative imaging protocols, specific to their systems.

Dual-compartment concentric spherical sources of accurately known activity concentrations were prepared and sent to seven European clinical sites. The site staff were not aware of the true volumes or activity within the sources—they performed SPECT/CT imaging of the source, positioned within a water-filled phantom, using their own choice of parameters and reported their estimate of the activities within the source.

**Results:**

The volumes reported by the participants for the inner section of the source were all within 29% of the true value and within 60% of the true value for the outer section. The activities reported by the participants for the inner section of the source were all within 20% of the true value, whilst those reported for the outer section were up to 83% different to the true value.

**Conclusions:**

A variety of calibration and segmentation methods were used by the participants for this exercise which demonstrated the variability of quantitative imaging across clinical sites. This paper presents a method to assess consistency between sites using different calibration and segmentation methods.

## Background

The accuracy of quantitative single photon emission tomography (SPECT) imaging is critical if the absorbed doses to organs or tumours are to be determined following administration of a radiopharmaceutical [13, 32]. However, for clinical use, SPECT has yet to be proved as a reliable quantitative tool, taking into account the different technical challenges. Guidance on quantitative SPECT has been issued by the European Association of Nuclear Medicine (EANM) [20], the International Atomic Energy Agency (IAEA) [18] and Medical Internal Radiation Dose (MIRD) Committee ([9, 10, 22]), including different methodologies of quantitative imaging. However, in most cases, these documents do not provide any direct recommendations. Many other publications have proposed a variety of methods for performing dosimetry when using radiopharmaceuticals in therapy ([26, 31, 35]), including the use of SPECT/CT measurements ([2, 4, 8, 12–14, 21, 23, 27, 28, 30, 36, 38, 39]). As a result, different centres use different techniques, which makes comparisons of any reported dosimetric results difficult. A multi-centre inter-comparison study has previously been performed using barium-133 (^133^Ba) as a surrogate for iodine-131 (^131^I), in order to compare the quantitative imaging results using various planar and SPECT imaging protocols [40]. This study demonstrated significant variability in quantitative accuracy between protocols but concluded that standardised protocols were needed to ensure consistent results.

Due to the increased interest in accurate dosimetry for molecular radiotherapy (MRT), the need to establish equivalence between sites is critical, particularly if large-scale multi-centre randomised clinical trials are to take place. In order to establish this equivalence and compare different techniques, the National Physical Laboratory (NPL) in the UK, along with several other National Metrology Institutes and clinical partners from across Europe, was awarded funding to address the metrology challenges associated with determining the radiation dose to patients undergoing MRT (EURAMET Project HLT11: Metrology for Molecular Radiotherapy). As part of this collaboration, a further inter-comparison exercise between a representative subset of seven European hospitals was carried out in order to establish the inter-hospital variability with regard to quantitative SPECT/CT imaging for ^177^Lu, a commonly used radionuclide for Peptide Receptor Radionuclide Therapy (PRRT) [3, 22]. The hospitals were provided with independently calibrated radioactive sources and phantoms and used their own choice of calibration, acquisition, reconstruction and image processing protocols.

The results of this inter-comparison will provide a means to demonstrate the variability in current clinical quantitative imaging methods, and establish the feasibility of international comparison exercises as a means to arrive at consistency whilst allowing clinical centres to use their own choice of calibration methods, specific to their systems.

## Methods

### Inter-comparison phantom

**Fig. 1:**
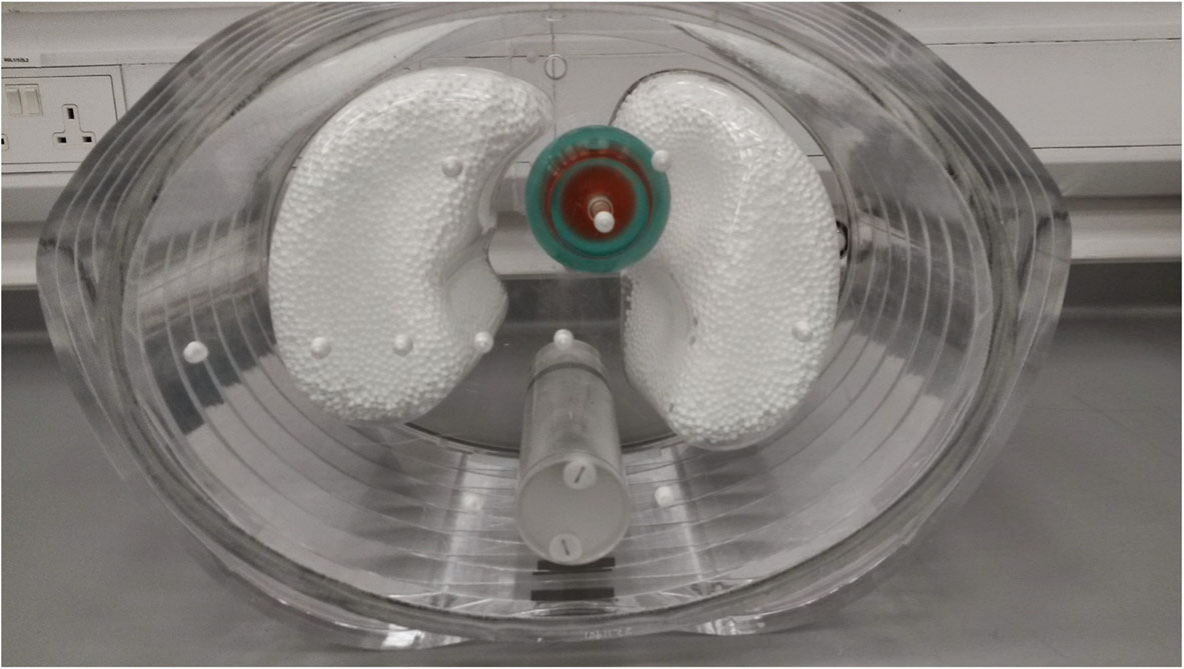
Comparison phantom shown in measurement setup prior to filling with water

**Fig. 2:**
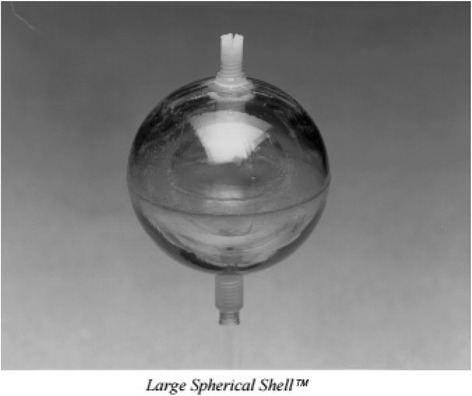
The DSC large spherical shell used in the comparison exercise

The inter-comparison exercise used six accurately calibrated radioactive sources (prepared at the NPL—the UK National Measurement Institute) and a single phantom. The phantom used is shown in Fig. 1 and was based on an elliptical Jaszczak phantom supplied by Data Spectrum Corporation (DSC, Durham, US) with lung and spine inserts and body contour rings added. The spine insert was filled with bone equivalent solution of dipotassium hydrogen orthophosphate (100 g dipotassium hydrogen phosphate dissolved in 67-g water [11]). The lungs were filled with Styrofoam© beads mixed with water with a resulting composition of 60% Styrofoam and 40% water by volume, resulting in a density of approximately 0.3 g/cm^2^ to mimic the lung tissue. The same phantom was used at each site, and no modifications were made to the contents of the lungs or spine insert between sites. The radioactive comparison sources consisted of a ‘shell sphere’ (also provided by DSC) which is shown in Fig. 2 and consist of two isolated concentric spheres: an ‘inner sphere’ of nominal diameter 36 mm filled with a high activity concentration, surrounded by a less-active ‘outer shell’ of nominal thickness 11 mm. The measured volumes of the shell spheres are presented in Table 1. Six different comparison sources were prepared at NPL and shipped to the sites directly, immediately prior to the inter-comparison visit, such that an approximately equal amount of activity was measured at each site. Table 1 shows the detailed dimensions of the phantom and its components, including shell spheres. The small differences in the shell constructions are estimated to have a negligible influence on the final results.

**Table 1:** Comparison phantom component specifications

Elliptical Jaszczak (ECT/ELP/P)				Body contour rings (ECT/BCR)		
Internal diameter (major axis)	Internal diameter (minor axis)	Internal height	Wall thickness	External diameter (major axis)	External diameter (minor axis)	Thickness (per ring)
305 mm	221 mm	186 mm	64 mm	380 mm	260 mm	25 mm
Lung/spine insert (ECT/LUNG/I)						
Fillable spine					Lungs	
Internal diameter	Internal length	External diameter	External length	Internal volume	RH lung volume (incl. Styrofoam beads)	LH lung volume (incl. Styrofoam beads)
38 mm	152 mm	45 mm	190 mm	170 ml	1100 ml	900 ml
Shell spheres (ECT/SPS-LG/A)						
	H1	H2	H3	H4 and H5	H6	H7
Inner sphere volume (ml)	26.1	26.0	26.3	26.7	26.0	27.2
Outer shell volume (ml)	80.8	81.3	80.8	79.3	81.6	80.7

The choice of inter-comparison phantom was driven by a desire to use an off-the-shelf pseudo-anthropomorphic set-up approximately simulating an active lesion in an organ of lower activity that could be readily replicated by other users (i.e. using commercially available phantoms). Ideally, the background volume would have been larger than described here, but this was limited by the practicalities of transporting the sources to the participating sites.

### Phantom and source preparation

Each source was prepared at NPL by the standardisation of stock solutions at two activity concentrations (to represent a ‘hot’ lesion in a ‘warm’ background/organ) at a ratio of approximately 15:1. Ampoules and vials from each stock solution were analysed on two independent secondary standard ionisation chamber systems which had been previously calibrated directly against primary standards by NPL and by a well-calibrated secondary standard High-Purity Germanium (HPGe) spectrometer to ensure no radionuclidic impurities were present. The activity content of the sources were chosen such that no dead-time correction would be required by any of the sites and that the source could be measured within a realistic time frame. A carrier solution was used to prepare each of the stock solutions and was comprised of 0.1 M hydrochloric acid solution also containing 10 μg g^−1^ inactive lutetium. The empty phantom spheres were first treated, prior to filling with radioactivity, for a minimum of 24 h with inactive carrier solution to ensure activity did not adhere to the walls of the shells [25].

The inactive parts of the phantom were transported to each site by NPL and were assembled with the active shell sphere before being filled with inactive water, excluding as many air bubbles as possible, and sealed. The sources were reproducibly located within the phantom by the use of threaded rods. The phantom was placed on absorbent material on the imaging couch in the ‘head first’ position and centred between the detector heads.

### Phantom measurement

A SPECT/CT image of the phantom was acquired by each of the sites, using their own choice of quantitative imaging protocol. Every site corrected the data for attenuation using the acquired CT data, most sites corrected the data for scatter (techniques detailed in the relevant sections), and some sites also corrected for collimator-detector response using resolution recovery methods [34]. The acquisition and processing parameters are summarised in Table 2 in addition to being detailed in the text below.

**Table 2:** Summary of acquisition and processing parameters used. See text for full details

	Site						
	1	2	3	4	5	6	7
Camera	Siemens Symbia T2	Siemens Symbia T2	GE Infinia Hawkeye 4	Siemens Intevo	Ge Optima 640	Siemens Symbia T6	GE Discovery 670
Collimator	ME	ME	MEGP	ME	MEGP	MELP	MEGP
Crystal thickness	5/8″	3/8″	3/8″	3/8″	3/8″	3/8″	5/8″
Photopeak(s), keV	–	113 ± 7.5%	113 ± 10%	113 ± 10%	–	–	–
	208 ± 10%	208 ± 10%	208 ± 10%	208 ± 10%	208 ± 10%	208 ± 10%	208 ± 10%
Scatter window(s), keV	–	–	–	98.7 ± 5%	–	–	–
	–	–	–	131 ± 5%	–	–	–
	176.8 ± 5%	–	–	178 ± 5%	178 ± 5%	–	–
	239.2 ± 5%	–	–	214 ± 5%	214 ± 5%	–	–
Orbit type	Contoured	Circular	Contoured	Contoured	Contoured	Circular	Contoured
Matrix	128 × 128	128 × 128	128 × 128	128 × 128	128 × 128	128 × 128	128 × 128
Number of projections	120	120	120	120	120	120	120
Time per projection	30 s	30 s	30 s	30 s	30 s	30 s	60 s *
Reconstruction type	OSEM	OSEM	OSEM	OSEM	OSEM	OSEM	OSEM
Iterations/subsets	6/6	8/4	16/5	24/24	5/10	5/15	8/10
Attenuation correction	CT	CT	CT	CT	CT	CT	CT
Scatter correction	TEW	No	Monte Carlo	TEW	TEW	Monte Carlo	ESSE
Resolution recovery	Yes	No	Yes	Yes	No	Yes	Yes
Segmentation method	CT VOI + 10 mm	SPECT threshold	CT VOI	CT VOI	CT VOI	CT VOI	CT VOI plus SPECT auto-threshold

*The activity of the source used at site H7 was approximately 50% of that used in all other sites, so the acquisition time was doubled to compensate for this

Each site had completed SPECT/CT quality assurance procedures including uniformity, energy peaking and CT/NM registration prior to the phantom imaging, according to their local protocols.

#### Hospital 1—Siemens Symbia T2 (5/8″ crystal)

##### Camera calibration and acquisition parameters

A 130**-**ml plastic bottle and a NEMA body phantom containing three hollow glass spheres (PTW, Freiburg, Germany) were used. The three largest spheres (internal diameters 22, 28 and 37 mm) and the bottle were filled with a homogeneous activity solution of 2.0 MBq/ml ^177^Lu-DOTATATE. The phantom was filled with water. The plastic bottle was positioned immediately adjacent to the NEMA phantom and a SPECT/CT image acquired. The same acquisition parameters were used for calibration and the inter-comparison exercise (medium energy collimators, contoured orbit, 128 × 128 matrix, 120 projections, 30 s per projection, photopeak 208 ± 10% keV, scatter windows of 176.8 ± 5% and 239.2 ± 5% keV). Volumes of interest (VOIs) were drawn around the bottle and the three active spheres on the CT data as the physical volume plus 10 mm (in each spatial direction).

The cps/MBq factor was obtained from the bottle VOI. The activities within each sphere were assessed to establish the volume below which partial volume corrections should be applied.

##### Image reconstruction

The projection data was reconstructed using reconstruction software provided by the camera manufacturer, using an ordered subset expectation maximisation (OSEM) algorithm with 6 iterations and 6 subsets, with triple energy window (TEW) scatter correction, CT attenuation correction and resolution recovery enabled.

##### Image segmentation

The inner sphere was segmented using a spherical VOI aligned to the CT images, with the sphere diameter set to be the physical diameter of the sphere (as measured on the CT) plus 10 mm.

The outer shell was segmented using a spherical volume aligned to the CT data, with the sphere diameter set to be the physical diameter of the external surface of the outer shell plus 10 mm. The VOI of the inner sphere was then subtracted from the VOI of the external surface of the outer shell to obtain the data for the shell. The arbitrary expansion of the region of interest by 10 mm in each direction is the standard clinical protocol at this site as it is felt that this will allow for correction for spill-out due to partial volume effects. This method therefore resulted in an 11-mm-thick outer shell VOI that only included the outermost 1 mm of the physical outer shell volume, and the rest of the physical outer shell volume was included in the inner sphere VOI.

##### Uncertainty estimation

The standard deviation in counts (Poisson noise) in the calibration measurement combined in quadrature with the uncertainty in the activity concentration of the ^177^Lu solution. No estimate of the uncertainty in the volumes was provided.

#### Hospital 2—Siemens Symbia T2 (3/8″ crystal)

##### Camera calibration and acquisition parameters

A shell sphere of the same design as that used for the inter-comparison was filled with a known quantity of ^177^Lu-DOTATATE and imaged in a cylindrical water-filled phantom. The same acquisition parameters were used for calibration and the inter-comparison exercise (medium energy collimators, circular orbit, 128 × 128 matrix, 120 projections, 30 s per projection, photopeaks 113 ± 7.5%, 208 ± 10% keV).

^177^Lu-DOTATATE was first added to the outer shell only (with the inner sphere empty) and the phantom imaged. Then, ^177^Lu-DOTATATE was added to the inner sphere, and the phantom was imaged again. The same activity concentration was used for both the inner sphere and the outer shell. These two images were used to select appropriate thresholds for the image segmentation.

Further images were then acquired with the shell sphere located at various depths within the cylindrical phantom in order to determine the cps/MBq factor for the source at each of the different depths within the phantom. This yielded a number of cps/MBq factors, and the factor for the calibration source at the most similar depth to the position of the inter-comparison source was used to obtain an activity value for the inter-comparison source.

##### Image reconstruction

The projection data was reconstructed using reconstruction software provided by the camera manufacturer, using OSEM with 8 iterations and 4 subsets, with CT attenuation correction. No scatter correction or resolution recovery was applied. The two sets of photopeak data were reconstructed individually (with CT attenuation maps generated for each photopeak) and then summed after reconstruction.

##### Image segmentation

An iso-contour of 35% was used to segment the surface of the inner sphere and 10% to segment the external surface of the outer shell on the SPECT data. The VOI of the inner sphere was subtracted from the outer shell external surface to obtain the data for the outer shell. The iso-contour thresholds were determined by varying the thresholding value to obtain a volume that most closely matched the known volumes of the shells.

##### Uncertainty estimation

The standard deviation in calibration factors calculated for each of the calibration images. No estimate of the uncertainty in the volumes was provided.

#### Hospital 3—GE Infinia Hawkeye 4 (3/8″ crystal)

##### Camera calibration and acquisition parameters

A 16-ml plastic sphere filled with approximately 20 MBq ^177^Lu with no radionuclidic impurities was imaged in the centre of a water-filled elliptical Jaszczak phantom using the same acquisition parameters as used in the inter-comparison exercise (medium energy collimators, contoured orbit, 128 × 128 matrix, 120 projections, 30 s per projection, photopeaks 113 ± 10% and 208 ± 10% keV).

A VOI was drawn on the sphere on the CT images and transferred to the SPECT data to obtain the cps/MBq factor.

##### Image reconstruction

The 113- and 208-keV photopeak projection data were reconstructed in a third-party vendor-neutral reconstruction software using OSEM with 16 iterations and 5 subsets, with Monte Carlo-based scatter correction based on CT density information [29], with CT attenuation correction and resolution recovery enabled. The two sets of photopeak data were reconstructed individually (with CT attenuation maps generated for each photopeak) and then summed after reconstruction.

##### Image segmentation

The inner sphere and external surface of the outer shell were segmented using a spherical VOI drawn on the CT images. The VOI of the inner sphere was then subtracted from the VOI of the outer shell external surface to obtain the data for the outer shell.

##### Uncertainty estimation

The standard deviation in counts (Poisson noise) in the calibration measurement combined in quadrature with the uncertainty in the activity concentration of the calibration sphere. No estimate of the uncertainty in the volumes was provided.

#### Hospital 4—Siemens Symbia Intevo 16 (3/8″ crystal)

##### Camera calibration and acquisition parameters

A 16-ml plastic sphere filled with approximately 35 MBq ^177^Lu with no radionuclidic impurities was imaged in an elliptical Jaszczak phantom in three positions (in air at the centre of the phantom, in water at the centre of the phantom and in water at a 15-cm displacement from the centre of the phantom). The same acquisition parameters were used for calibration and the inter-comparison exercise (medium energy collimators, contoured orbit, 128 × 128 matrix, 120 projections, 30 s per projection, photopeaks 113 ± 10% and 208 ± 10% keV, scatter windows 98.7 ± 5%, 131 ± 5%, 178 ± 5% and 214 ± 5% keV). VOIs were drawn freehand by the operator on the CT images.

The cps/MBq factor was calculated as the mean of the cps/MBq value calculated for each of the three imaging positions.

##### Image reconstruction

The projection data was reconstructed using the reconstruction software provided by the camera manufacturer, using OSEM with 24 iterations and 24 subsets, with scatter correction, attenuation correction and resolution recovery enabled. Scatter correction was performed using TEW algorithm. The two sets of photopeak data were reconstructed individually (with CT attenuation maps generated for each photopeak) and then summed after reconstruction.

##### Image segmentation

VOIs were drawn freehand by the operator on the inner sphere and outer shell walls on the CT data.

##### Uncertainty estimation

The mean standard deviation in counts (Poisson noise) in the calibration volumes was combined in quadrature with the standard deviation in counts in the individual volumes of interest in the inter-comparison images. No estimate of the uncertainty in the volumes was provided.

#### Hospital 5—GE Optima 640 (3/8″ crystal)

##### Camera calibration and acquisition parameters

A 16-ml plastic sphere filled with approximately 25 MBq ^177^Lu with no radionuclidic impurities was imaged three times in an elliptical Jaszczak phantom at three different positions (in air at the centre of the phantom, in water at the centre of the phantom and in water at a 15-cm displacement from the centre of the phantom). The same acquisition parameters were used for calibration and the inter-comparison exercise (medium energy collimators, contoured orbit, 128 × 128 matrix, 120 projections, 30 s per projection, photopeak 208 ± 10% keV, scatter windows of 178 ± 5% and 214 ± 5% keV). Spherical VOIs were drawn on the CT images and transferred to the SPECT data. Since volumes on the reconstruction workstation only include whole pixels, two VOIs were drawn on each image: one slightly smaller than the physical size of the sphere and one slightly larger, resulting in 6 VOIs for three images.

The cps/MBq factor was calculated as the mean of the cps/MBq value measured for each of the six VOIs.

##### Image reconstruction

The projection data was reconstructed using the reconstruction software provided by the camera manufacturer, using OSEM with 5 iterations and 10 subsets, with attenuation correction enabled. The projection data was corrected for scatter prior to reconstruction, using an in-house TEW algorithm. Resolution recovery was not enabled.

##### Image segmentation

The inner sphere and external surface of the outer shell were segmented using spherical VOIs drawn on the CT images. The VOI of the inner sphere was then subtracted from the outer shell external surface VOI to obtain the data for the shell.

##### Uncertainty estimation

The standard deviation in the six calibration measurements combined in quadrature with the uncertainty in the activity concentration of the calibration sphere. No estimate of the uncertainty in the volumes was provided.

#### Hospital 6—Siemens Symbia T16 (3/8″ crystal)

##### Camera calibration and acquisition parameters

A 6.9-l cylindrical Jaszczak phantom containing a homogenous solution of approximately 120 MBq of Lu^177^-DOTATATE was imaged. The same acquisition parameters were used for the calibration and the inter-comparison exercise (medium energy collimators, circular orbit, 128 × 128 matrix, 120 projections, 30 s per projection, photopeak 208 ± 10% keV).

A cps/MBq factor was determined using a large VOI positioned centrally within the phantom.

##### Image reconstruction

The projection data was reconstructed in a third-party vendor-neutral reconstruction software using OSEM with 5 iterations and 15 subsets, with scatter correction, attenuation correction and resolution recovery enabled. Scatter correction was performed using Monte Carlo simulations based on the CT density information [29]. A Gaussian filter with a Full Width at Half Maximum of 8 mm was applied post-reconstruction.

##### Image segmentation

The inner sphere was segmented using a spherical VOI aligned to the CT images, with the sphere diameter set to 8 cm. No results were reported for the outer shell.

##### Uncertainty estimation

No estimation of uncertainty was performed as the calibration performed by this site was done using previously acquired data that did not have any associated uncertainty values.

#### Hospital 7—GE Discovery 670 (5/8″ crystal)

##### Camera calibration and acquisition parameters

Five stock vials, each containing approximately 40 MBq of Lu^177^-DOTATATE in a 4-ml solution, were made. These were then dispensed into five petri dishes, each 6 cm in diameter, and planar images were acquired for each camera head, at 10-cm distance from the collimator. The counts in a circular region of interest (ROI) with a diameter of 10 cm drawn around the petri dish on each image were obtained. The diameter was set at 10 cm, as previously determined by gradually increasing the diameter until it encompassed the counts contribution from the source. ROIs were also drawn outside the petri dish to assess the number of counts in the background.

The cps/MBq factor was calculated as the mean of the cps/MBq value calculated for each of the five sources.

A recovery-coefficient curve was also determined, by obtaining SPECT images of various sized spheres with known activity. The same acquisition parameters were used for the recovery curve imaging and the inter-comparison exercise (medium energy collimators, contoured orbit, 128 × 128 matrix, 120 projections, 60 s per projection, photopeak 208 ± 10% keV). An auto-contouring algorithm, based on the Otsu method [24], was used to delineate the spheres, and a curve was fitted to the recovery coefficient versus sphere volume data.

##### Image reconstruction

The projection data was reconstructed in an in-house vendor-neutral reconstruction software using OSEM, with 8 iterations and 10 subsets, with scatter correction, attenuation correction and resolution recovery enabled. Scatter correction was performed using effective source scatter estimation (ESSE) method [15], using Monte Carlo-calculated scatter kernels as input. The previously determined recovery coefficient equation was then applied to the result to obtain the activity concentration in the inner sphere.

##### Image segmentation

The inner sphere was segmented on the SPECT data using an auto-contouring algorithm based on the Otsu method [24]. The outer shell was segmented manually by the operator on the CT data. The estimated activity in the inner sphere was then subtracted from the VOI encompassing the entire shell sphere to give the data for the outer shell.

##### Uncertainty estimation

The standard deviation in the five calibration measurements combined in quadrature with previously estimated uncertainties of recovery coefficients. No estimate of the uncertainty in the volumes was provided.

## Results

Participants reported results independently following the completion of the exercise and were assigned unique identifiers to maintain confidentiality of the results.

### Volume

**Fig. 3:**
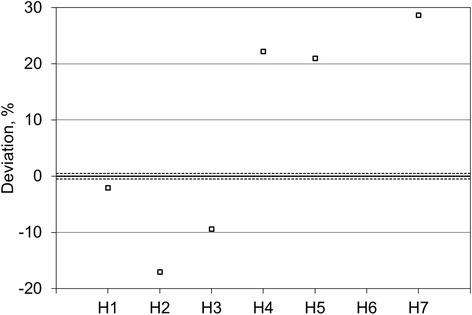
Participants reported volumes for the inner sphere of the comparison source. Since H6 used an arbitrary spherical volume of 8 cm in lieu of outlining the inner sphere, no volume is included in this graph

Figure 3 shows the volumes reported by six of the sites for the inner sphere of the comparison source, given as the difference between the locally measured volume and the true volume, as measured at NPL. The data shows a spread of 46% (range − 17 to + 29%) with a slight tendency to overestimate the volume by a mean value of 7%. The two sites that segmented using auto-contouring technique on the SPECT data (H2 and H7) reported the two answers furthest from the truth (− 17 and + 29% difference to the true value, respectively). Sites that segmented using the CT data reported answers between − 9 and + 22% from the true value of the volume.

**Fig. 4:**
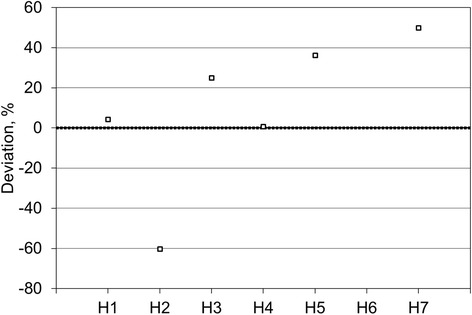
Participants reported volumes for the outer shell of the comparison source

Figure 4 shows the volumes reported by six of the sites for the outer shell of the comparison source, given as the difference between the locally measured volume and the true volume, as measured at NPL. The data shows a spread of 110% (range − 60 to + 50%) with a tendency to overestimate the volume by a mean value of 9%. As for the inner sphere, the two sites that segmented at least partially using auto-contouring technique on the SPECT data (H2 and H7) reported the two answers furthest from the truth (− 60 and + 50% difference to the true value, respectively). Sites that segmented entirely using the CT data reported answers between + 1 and + 36% from the true value.

**Fig. 5:**
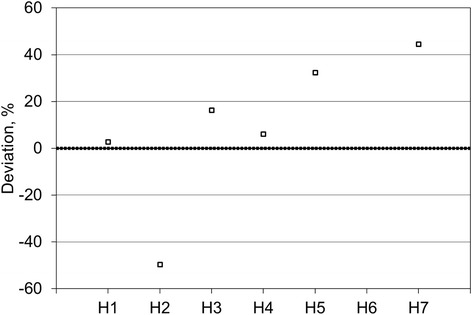
Participants reported volumes for the entire comparison source

Figure 5 shows the volumes reported by six of the sites for the volume of the entire comparison source, given as the difference between the locally measured volumes and the true volumes, as measured at NPL. The data shows a spread of 95% (range − 50 to + 45%), with a tendency to overestimate the volume by a mean value of 9%.

H1 reported volumes within 5% of the true value for both parts of the comparison source (inner sphere − 2%, outer shell 4%).

### Activity

**Fig. 6:**
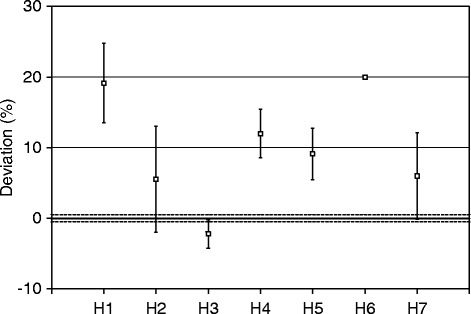
Participants reported activities for the inner sphere of the comparison source

Figure 6 shows the results reported by the sites for the inner sphere of the comparison source, given as the difference between the locally measured activity values and the true activity, as calibrated at NPL. The data shows a spread of 22% (range − 2 to + 20%) with a tendency to overestimate the activity by a mean value of 10%. H3 reported the result that was most consistent with the NPL value for the inner sphere and also had the lowest uncertainty of the participants. Three of the participants’ (H2, H3 and H7) reported ranges included the NPL value. The remaining four results were within 20% of the NPL value.

**Fig. 7:**
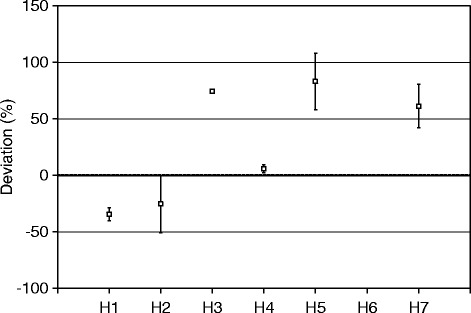
Participants reported activities for the outer shell of the comparison source

Figure 7 shows the results reported by the sites for the outer shell of the comparison source, given as the difference between the locally measured activity values and the true activity, as calibrated at NPL. The data shows a spread of 117% (range − 34 to + 83%), with a tendency towards overestimating the activity by a mean value of 27%. H4 reported the closest result to the NPL value, but the reported range did not include the NPL result. Only H2 reported a range that included the NPL value. Three of the participants’ (H1, H2, H4) results were within 50% of the NPL value, with all results being within 100%.

**Fig. 8:**
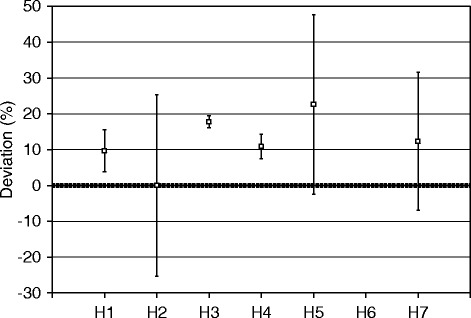
Participants reported activities for the entire comparison source

Figure 8 shows the results reported by the sites for the total activity of the comparison source (the sum of the activity for the inner sphere and the outer shell), given as the difference between the locally measured activity values and the true activity, as calibrated at NPL. The data shows a spread of 23% (range 0 to + 23%), with all participants overestimating the total activity by a mean value of 12%. Three of the participants’ (H2, H5 and H7) reported ranges included the NPL value and all results were within 30% of the NPL value.

Table 3 shows a breakdown of the agreement between locally measured absolute activity values for the comparison source compared to the true activity. Table 4 shows a breakdown of the agreement between the locally measured activity ranges for the comparison source compared to the true activity.

**Table 3:** Proportion of sites for which reported values were within the given percentage of the true value

	Within 5%	Within 10%	Within 20%	Within 50%	Within 75%	Within 100%
Inner sphere	1/7 (14%)	4/7 (57%)	7/7 (100%)	7/7 (100%)	7/7 (100%)	7/7 (100%)
Outer shell	0/6 (0%)	1/6 (17%)	1/6 (17%)	3/6 (50%)	5/6 (83%)	6/6 (100%)
Total source	1/6 (17%)	2/6 (33%)	5/6 (83%)	6/6 (100%)	6/6 (100%)	6/6 (100%)

**Table 4:** Proportion of sites for which reported uncertainty on the measured activity was within the given percentage of the true value. Since H6 did not report an uncertainty, the data from H6 has been excluded from this table

	Within 5%	Within 10%	Within 20%	Within 50%	Within 75%	Within 100%
Inner sphere	3/6 (50%)	5/6 (83%)	6/6 (100%)	6/6 (100%)	6/6 (100%	6/6 (100%)
Outer shell	2/6 (33%)	2/6 (33%)	2/6 (33%)	4/6 (67%)	6/6 (100%)	6/6 (100%)
Total source	4/6 (67%)	5/6 (83%)	6/6 (100%)	6/6 (100%)	6/6 (100%)	6/6 (100%)

### Effect of volume on activity

For all sites that used a non-specific calibration method (i.e. all sites other than H2), the use of a VOI larger than the true volume of the inner sphere resulted in a reported activity higher than the true value and likewise for VOIs smaller than the true volume. The same was true for the outer shell, with the exception of H1, for which the segmentation method (expanded volume: expanded on outer surface of the shell but reduced on inner surface of the shell) meant that the majority of the outer shell volume was included in the inner sphere VOI rather than the outer shell.

## Discussion

Different participants used different segmentation methods depending on whether they used the knowledge that the comparison source was spherical and drew spherical VOIs on the CT (H1, H3, H5 and H6) or whether they assumed no prior knowledge and segmented the source manually (H4 and H7) and/or using thresholding techniques (H2 and H7). The resultant volumes indicate that segmenting on the SPECT data is likely to be less accurate than segmenting on CT data, regardless of whether prior knowledge of the shape of the VOI is utilised. The use of VOIs larger than the physical volume results in reported activity higher than the true value and vice versa, since this will result in the inclusion of additional voxels (for larger VOIs) or exclude voxels that should be included (for smaller VOIs). The uncertainty in the final activity is therefore strongly dependent on the uncertainty in the segmentation method used, but none of the participants included an estimate of the segmentation uncertainty in their activity uncertainty estimation.

The overestimation of activity for the inner sphere may also be partly due to the inclusion of ‘spill in’ from the warm (1:15) outer shell. It was not anticipated that the inner sphere would have suffered significantly from ‘spill out’ since it is a volume of 26 ml (internal diameter 36 mm), which is approximately equivalent to the largest fillable spheres used by clinical sites to measure partial volume effects [6, 7]. As part of the calibration measurements, H1 determined that partial volume corrections were not necessary for this volume on their Siemens gamma camera when resolution recovery is used. However, recent simulation work [16] for a GE gamma camera found that partial volume effects are less than 15% at this size if resolution recovery is performed (H1, H3, H4, H6 and H7). The same study found that if resolution recovery is not used (H2 and H5), partial volume corrections of less than 30% are likely to be required. Furthermore, four of the participants used sphere of similar or smaller volumes than that of the inner sphere (H1, H3, H4 and H5) to calibrate their systems, which will have resulted in some degree of partial volume correction being incorporated into their cps/MBq factors (if partial volume corrections were necessary at these volumes).

However, due to its smaller thickness (11 mm), the outer shell was more likely than the inner sphere to have been subject to significant partial volume effects due to both image sampling (pixelation) and image blurring (low spatial resolution). Only one participating site corrected for partial volume effects (H7) whilst five of the sites used resolution recovery (H1, H3, H4, H6 and H7). As already noted, the activity concentrations in the outer shell were relatively low compared to the inner sphere. There was thus significantly increased Poisson noise in the outer shell in comparison to the inner sphere due to low counting statistics which may also have contributed to the observed deviations. Three of the sites (H1, H3 and H4) accounted for Poisson noise in their uncertainty estimation.

As mentioned in the ‘Methods’ section, the shell sphere chosen for this exercise was selected for logistical purposes—the inclusion of background activity was desired, but the source had to be able to be prepared at NPL and shipped to the participants. It was unfeasible to transport a 9-l phantom filled with liquid activity, and shipping vials of activity to be added to the water in the phantom at each site would have contaminated the phantom, preventing onward transportation of the phantom to the next site. The shell source selected was commercially available, and it was possible to obtain transportation containers that made shipping them a viable option. If this comparison exercise were to be repeated with a similar set-up, it may be desirable to use a shell sphere with a thicker outer shell.

When the activity of the inner sphere and the outer shell was summed to give a total source activity (Fig. 8), the participants still overestimated the activity in the source but by substantially less than what was reported for the outer shell. Summing the activity in the inner sphere with that in the outer shell ensures that some of the spill-out from the outer shell is included. This result highlights the importance of accurate VOI drawing and performing partial volume correction to exclude spill-out from neighbouring active regions.

^177^Lu has two main gamma emissions [1], at 113 keV (6%) and 208 keV (10%). Three participants (H2, H3, H4) chose to acquire both photopeaks to maximise the total number of counts acquired, whilst four participants (H1, H5, H6, H7) acquired only the 208-keV photopeak in order to reduce scatter within the image [17]. It should be noted that the choice of photopeaks at each site was determined by standard clinical practice and was not tailored to this specific set-up. In this set-up, scatter is likely to be much less significant than in, for example, a patient with a large lesion located close to a lower activity organ of interest. Our data showed no advantage to using 208 keV in comparison to using both 113 and 208 keV in this situation, but other variations in the protocols used by each participant make it impossible to draw any conclusions.

Two sites (H1 and H7) had SPECT/CT systems with 5/8″ crystals, compared to the 3/8″ for all other sites and a thicker crystal will provide greater sensitivity at the cost of a reduction in spatial resolution. As with the choice of photopeaks, our data showed no advantage to using 5/8″ over 3/8″, but the variations in the techniques used make it impossible to draw any conclusions.

^177^Lu can be produced with or without the addition of a carrier [5]. The manufacturing method for carrier-added ^177^Lu results in low-level contamination of the final product with ^177m^Lu—typically around 0.01–0.02% at the end of irradiation [5], whereas the non-carrier-added (n.c.a.) method results in no ^177m^Lu impurities in the final product. The comparison source was filled with n.c.a. ^177^Lu, and H3, H4 and H5 performed their calibrations using n.c.a. ^177^Lu whilst H1, H2, H6 and H7 used ^177^Lu-DOTATATE which was likely to contain trace amounts of ^177m^Lu. ^177m^Lu has a 160-day half-life, and so as long as the calibration measurements were done reasonably promptly after production, the presence of any ^177m^Lu impurities would have been negligible and our data showed no benefit to using n.c.a. ^177^Lu rather than ^177^Lu-DOTATATE.

The calibration geometries used at different sites varied from using SPECT/CT imaging of a comparably large homogenous source (H1, H6), a homogenous spherical 16-ml source (H3, H4, H5) or a shell sphere of the same design as the comparison source (H2) to planar imaging of activity in a petri dish (H7). Results do not clearly point at one method being superior to the others. The option of using the same geometry for calibration and imaging (H2) is not currently available in clinical studies, although some research groups have proposed that 3D printing of patient-specific phantoms is potentially feasible [33, 37] and may be the future of MRT dosimetry. A limitation of 3D printing patient-specific phantoms is the inability to model inhomogeneous activity uptake (although multi-compartment models can be made allowing a number of different activity concentrations to be used). However, the speculation is that since many other image properties will be the same at calibration and imaging (such as scatter, attenuation and volume, position, shape and surface area of VOI), patient-specific calibrations could provide more accurate results by removing the need to extrapolate from calibration measurements to volumes of vastly differing properties. In this small study, the site that used this method (H2) was the only site to report activity ranges including the true NPL activity values for both the inner sphere and outer shell.

Some issues were encountered with the study due to different participants interpreting the task in different ways. These issues could have been addressed had the instructions been clearer and the various possible interpretations considered when designing the comparison. Different participants used different segmentation methods depending on whether they used the knowledge that the comparison source was spherical, and drew spherical VOIs on the CT (H1, H3, H5, H6), or whether they assumed no prior knowledge and segmented the source manually (H4, H7) and/or using thresholding techniques (H2, H7). Guidance could have been provided to the participants regarding this. However, the choice of segmentation method(s) will also affect the quantitation of the images, so it is interesting to include in this paper, and guidance was deliberately kept to a minimum to allow participants to use their own choice and judgement.

The outer shell was referred to as the ‘background region’ on the reporting form, and there was some confusion as to what part of the phantom that meant. As a result, H6 reported the background region as a VOI located in a non-active region of the phantom rather than the outer shell. Clearer naming of the parts of the comparison source would have prevented this from happening.

The results also show that a great deal of work is still required to reduce the uncertainties in quantitative SPECT to enable individual patient doses to both tumours and surrounding healthy tissue to be calculated within limits generally considered acceptable in external beam radiotherapy (within 5% [19]).

## Conclusions

Participants described a variety of methods used to determine the cps/MBq factor, mostly centred on the measurement of a simple sphere in a Jaszczak phantom; however, no single method was identified as yielding significantly improved accuracy compared to the others due mostly to the small number of participants. Reasonable uncertainties were reported by some of the participants, and various methods were used to determine these uncertainties; however, further research into the sources of uncertainty should be performed in order to fully determine a realistic uncertainty budget. This inter-comparison only investigated a simple geometry, and corrections for partial volume effects, dead time or background concentration were not fully incorporated; however, the results reinforce the need for more guidance and present a method for assessing consistency in this area.
